# Structural analyses and substrate profiling of PPEP-3 provide new insights into the molecular basis of Pro-Pro endopeptidase specificity

**DOI:** 10.1016/j.isci.2025.114360

**Published:** 2025-12-08

**Authors:** Bart Claushuis, Fabian Wojtalla, Lisa Papenhagen, Robert A. Cordfunke, Arnoud H. de Ru, Hans C. van Leeuwen, Jeroen Corver, Paul J. Hensbergen, Ulrich Baumann

**Affiliations:** 1 Center for Proteomics and Metabolomics, Leiden University Medical Center, Leiden 2333 ZA, the Netherlands; 2 Department of Chemistry, Institute of Biochemistry, University of Cologne, 50674 Cologne, Germany; 3 Department of Immunology, Leiden University Medical Center, Leiden 2333 ZA, the Netherlands; 4 Department of CBRN Protection, Netherlands Organization for Applied Scientific Research TNO, Rijswijk 2280 GJ, the Netherlands; 5 Leiden University Center for Infectious Diseases, Leiden University Medical Center, Leiden 2333 ZA, the Netherlands

**Keywords:** biological sciences, biochemistry, structural biology, proteomics, methodology in biological sciences

## Abstract

Pro-Pro endopeptidases (PPEPs) are secreted bacterial enzymes that uniquely cleave peptide bonds between adjacent proline residues. Their active site accommodates six substrate residues (P3 to P3′), with interactions at these positions determining specificity. In this study, we investigated the substrate specificity of PPEP-3 from *Geobacillus thermodenitrificans* using synthetic peptide libraries and liquid chromatography-tandem mass spectrometry (LC-MS/MS). We also determined the atomic structures of PPEP-3 in unbound and substrate-bound forms. By correlating substrate profiling with structural data, we identified key mechanisms influencing PPEP-3 specificity. This integrated analysis reveals stark differences in specificity for the P2 and P2′ positions compared to other PPEPs, most notably Tyr161 and Phe191, which shape the substrate-binding cleft and influence the accommodation of side chains at these positions. Combining comprehensive substrate profiling with structural analyses offers a powerful approach to uncover the molecular basis of protease function.

## Highlights

• Atomic structure of Pro-Pro endopeptidase 3 in unbound and substrate-bound forms
• The substrate specificity of PPEP-3 differs from other PPEPs
• Analysis of the PPEP-3 structure reveals key determinants of PPEP-3 specificity

## Graphical abstract

**Figure:**
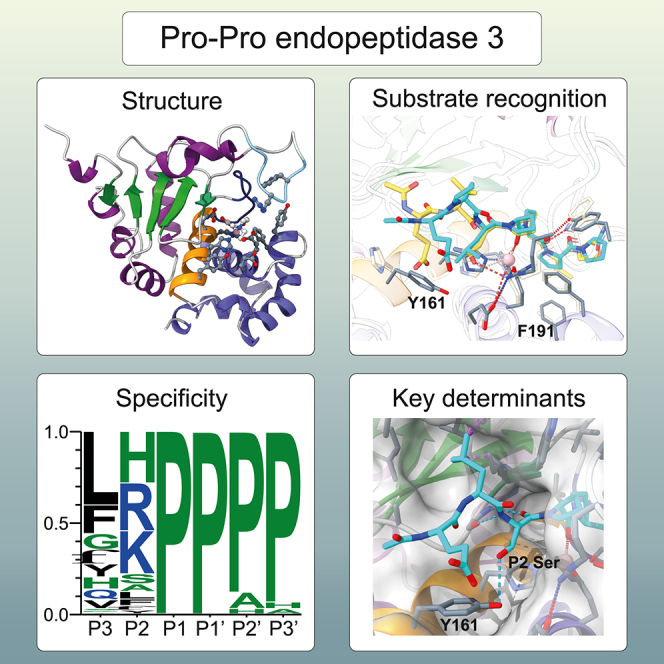

## Introduction

A group of bacteria have evolved a class of proteases with an unusual substrate specificity: the Pro-Pro endopeptidases (PPEPs). PPEPs are zinc metalloproteases characterized by the unique specificity to hydrolyze the peptide bond between two proline residues. PPEPs are extracellular proteases, either secreted in the environment or attached to the cell wall through additional domains.^1^ The first identified PPEP, PPEP-1 from the human pathogen *Clostridioides difficile*, acts as a switch between adhesion and motility by cleaving two adhesion proteins.^2^^,^^3^ This virulence factor has been used as a target in immunization studies, and anti-PPEP-1 antibodies reduce *C. difficile* pathogenesis.^4^ The second characterized PPEP, PPEP-2, is believed to play a similar role in *Paenibacillus alvei*.^5^ For both these PPEPs, the endogenous substrates are encoded by genes adjacent to the PPEP gene. In the case of two other PPEPs, PPEP-3 from *Geobacillus thermodenitrificans* (Uniprot: A4INY2) and PPEP-4 from the closely related organism *Anoxybacillus tepidamans* (Uniprot: A0A7W8IRZ3), no endogenous substrates or function have been identified so far.^6^^,^^7^ Interestingly, a PPEP homolog from *C. difficile*, CD1597, possesses a PPEP-like domain but exhibits no (Pro-Pro) proteolytic activity, suggesting potential divergence in function.^6^^,^^8^

Previously, atomic structures have been experimentally determined for PPEP-1 and PPEP-2.^5^^,^^9^^,^^10^^,^^11^ Overall, these structures display highly similar structural elements. The proteases consist of an N-terminal (NTD) and C-terminal domain (CTD), which are divided by an active site helix containing the HEXXH motif of metalloproteases.^5^^,^^9^ For PPEP-1, cocrystal structures in complex with substrate peptides have been resolved.^9^^,^^11^ In these cocrystals, the substrate binds in a double-kinked conformation produced by X-Pro bonds in the peptide.^9^ This conformation is required due to a structural element called the diverting loop, which otherwise restricts the substrate from exiting the active site cleft and therefore greatly impacts PPEP specificity.^9^ Another important structural feature is the flexible S-loop, which closes upon substrate binding and thereby covers a part of the active site cleft.^9^^,^^11^

The active site cleft of PPEPs accommodates the six substrate residues P3 to P3’ (with P1 and P1′ being Pro) according to the nomenclature developed by Schechter and Berger.^12^ Previously, we developed a method to characterize PPEP specificity in detail using synthetic combinatorial peptide libraries and liquid chromatography-tandem mass spectrometry (LC-MS/MS).^6^^,^^7^ Using this method, we previously profiled the complete substrate specificity for PPEP-1, PPEP-2, and PPEP-4,^6^ while for PPEP-3 only the prime-side specificity has been determined so far.^7^ A remarkable feature of the prime-side specificity of PPEP-3 is the preference for all prolines at the P1′-P3′ positions, whereas other PPEPs display more variability.^6^^,^^7^ For example, endogenous PPEP-1 and PPEP-2 substrates with a valine at the P2′ position are not cleaved by PPEP-3, while substitution of the P2′ residue with a proline allows for proteolysis by PPEP-3.^7^ A detailed understanding of the substrate specificity of PPEPs in combination with substrate-bound protease structures allows us to describe the structure-function relationship at an atomic level. However, to identify both the general mechanisms and the unique determinants of PPEP specificity, additional cocrystal structures are needed.

By employing synthetic combinatorial peptide libraries combined with LC-MS/MS analyses, we were able to characterize both the non-prime- and prime-side specificity of PPEP-3. Moreover, we also determined the atomic structure of PPEP-3 from *Geobacillus thermodenitrificans* in the unbound form and in complex with a substrate peptide.

By integrating comprehensive substrate specificity profiles with structural data, we have demonstrated a powerful approach to probing protease specificity at the atomic level. While our focus has been on PPEPs, this methodology is broadly applicable to other proteases and could significantly advance our understanding of enzyme-substrate interactions. Ultimately, these insights may aid in the development of novel antimicrobial strategies or the design of proteases with an engineered specificity.

## Results

### Profiling the substrate specificity of PPEP-3 using synthetic combinatorial peptide libraries

**Figure 1:**
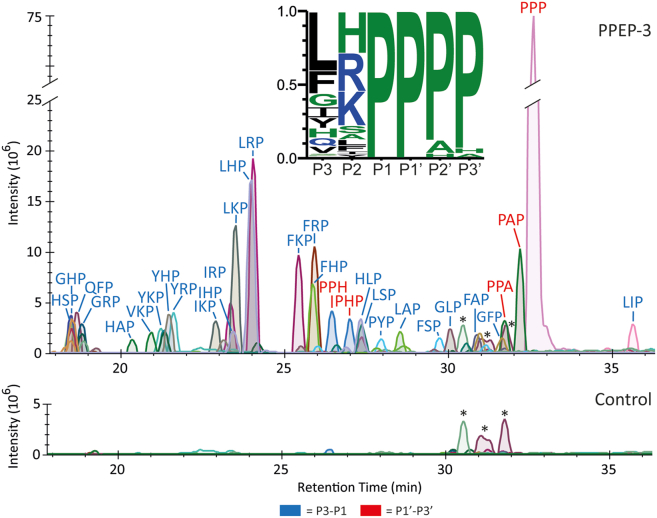
PPEP-3 specificity for amino acids surrounding the Pro-Pro cleavage site A combinatorial peptide library was incubated with PPEP-3, product peptides were analyzed by LC-MS/MS, and a database search was performed to identify and quantify the products. Results were filtered for 9-mer product peptides and the most abundant products that collectively account for >90% of the total abundance per library were used to create the EICs. The PTEDAVXXP (non-prime-side) and PXXGGLEEF (prime-side) product peptides are shown in blue and red, respectively. Mass tolerance was set to 5 ppm. An untreated control sample was included. A logo was created based on the product peptides to show the relative occurrence of the residue at a position surrounding the cleavage site. ∗MS/MS spectra did not indicate the presence of PTEDAVXXP or PXXGGLEEF product peptides.

We determined the non-prime- and prime-side specificity of PPEP-3 using synthetic combinatorial peptide libraries specifically designed for PPEPs.^6^ These libraries have two consecutive prolines in their core, while the surrounding positions are varied. We used two peptide libraries: one for determining the non-prime-side specificity and the other for the prime-side specificity. The non-prime-side library contains sequences with a PTEDAVXXPPXXEZZO motif (X = any residue except Cys, Z = 6-aminohexanoic acid, O = Lys(biotin)-amide). The prime-side library contains sequences with a JZEXXPPXXGGLEEF motif (X = any residue except Cys, Z = 6-aminohexanoic acid, J = biotin). The approach to profile the P3-P3′ specificity has been previously described.^6^ In short, the libraries were mixed and incubated with PPEP-3. Non-biotinylated product peptides originating from Pro-Pro cleavage (PTEDAVXXP at the non-prime-side or PXXGGLEEF at the prime-side) were enriched by negative selection on a streptavidin column and analyzed by LC-MS/MS. Extracted ion chromatograms (EICs) were produced showing the intensities of the product peptides (Figure 1), which showed a markedly different profile compared to other PPEPs (Figure S1^6^). Based on these intensities, a logo was constructed that shows the relative occurrence of a residue at a position surrounding the cleavage site (Figure 1).

We inspected the MS2 spectra to correctly annotate any ambiguous signals in the EIC. Based on the MS2 spectra alone, we were not able to discriminate between the isomeric residues Leu and Ile. However, the same peptide with Ile instead of Leu at a certain position tends to elute earlier^7^^,^^13^, enabling peptide assignment based on the retention time. Still, four signals were observed in the EIC, with one being much higher than the other. To discriminate between these for signals that originate from the PTEDAVIIP, PTEDAVLLP, PTEDAVLIP, and PTEDAVILP product peptides, we synthesized these four peptides and analyzed their retention on a C18 column using LC-MS/MS to annotate this signal. The isomeric peptides were completely resolved in time (Figure S2), allowing us to annotate PTEDAVLIP as the major signal in Figure 1.

### Atomic structure of PPEP-3

The crystal structure of PPEP-3 wild type was obtained at 1.55 Å resolution in the tetragonal space group P4_1_2_1_2 with two monomers per asymmetric unit (ASU). The structure of the inactive double mutant E154A/Y190F, which was produced for co-crystallization with substrate peptides, was determined at 1.60 Å in the same crystal form. The introduction of the double mutation did not lead to significant structural changes. On the other hand, the structure of the inactive mutant in complex with the peptide Ac-EPLPPPP-NH_2_, which was a known substrate of PPEP-3,^7^ was determined at a resolution of 2.02 Å in the space group P2_1_2_1_2_1_ with four monomers in the ASU. The data collection and refinement summary are presented in Table S1.

**Figure 2:**
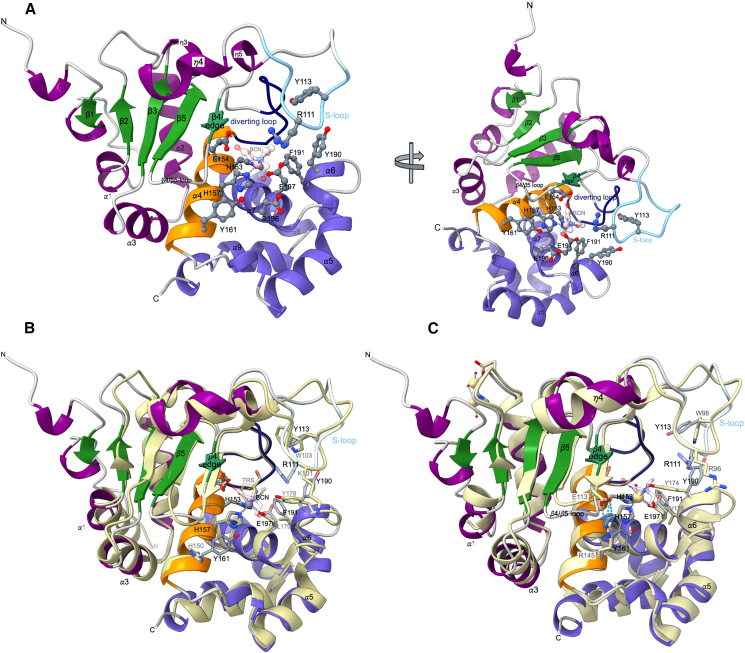
The overall structure of PPEP-3 and comparison with PPEP-1 and PPEP-2 (A) X-ray crystallographic structure of PPEP-3 in cartoon representation in two approximately orthogonal views. Shown are the N-terminal domain (NTD in green and purple, the active site helix (orange), the C-terminal domain (CTD) in slate blue, the S-loop in light cyan, the β4/β5-loop in gray, the diverting loop in navy blue, and the zinc ion in pink color. Zinc-coordinating and catalytically involved residue side chains are depicted as sticks. The two residues, Tyr161 and Phe191, which alter specificity compared to PPEP-1 at the P2 and P2′ sites are shown as well. A bicine molecule (BCN) is bound to the catalytic zinc ion. (B) Superposition of PPEP-3 (colors as in A) with PPEP-1 with a bound Tris buffer molecule (TRS) (pale goldenrod, PDB: 5N12, RMSD 1.4 Å). (C) Overlay of PPEP-3 (colors as in A) and PPEP-2 (pale goldenrod, PDB: 6FPC, RMSD 1.4 Å). In (B) and (C), residue numbers of PPEP-3 are in black, while those for PPEP-1 and PPEP-2 are in gray color.

PPEP-3 shares about 39% and 34% sequence identity with PPEP-1 and PPEP-2, respectively. The RMS deviations of equivalent C*α* atoms for the superposition of PPEP-3 with PPEP-1 or PPEP-2 are about 1.4 Å. Like for PPEP-1 and PPEP-2, the overall structure of PPEP-3 consists of an N-terminal domain (NTD) and a C-terminal domain (CTD) divided by the active-site helix carrying the HEXXH signature motif (Figure 2A). The active site helix α4 in PPEP-3 harbors the two histidine residues (His153 and His157) coordinating the catalytic zinc ion and the catalytic base Glu154, which collectively form the characteristic HEXXH motif of the zincin family.^14^

The α/β NTD consists mainly of the three *α*-helices α1-α3, and a five-stranded, mainly parallel β-sheet. The short and antiparallel β4 strand covers the active site helix *α*4 with the HEXXH motif. It is called the “edge strand” and serves to fix the substrate peptide segment in an extended conformation that runs antiparallel to it. A flexible loop, termed S-loop, interconnects helices η4 (a 3_10_ helix) and the edge strand via η5. In the substrate-unbound crystal form of PPEP-3 wild type and the E154A/Y190F double mutant, both crystallographically independent molecules exhibit well-resolved S-loops in an open conformation, similar to the open, substrate-free conformations of PPEP-1 (PDB: 5A0P, 5N12; chain B) and PPEP-2 (PDB: 6FPC) (Figures 2B and 2C).

PPEP-3 differs from PPEP-1 and PPEP-2 by an elongated N-terminus, which besides helix *α*1 contains two 3_10_-helical segments and the short strand β1 (Figures 2B, 2C, and S3). Additional important differences are observed in the active site of PPEP-3. In PPEP-1, the side chain of His150, which is lining the S2 pocket, is pointing out of the active site cleft in all known PPEP-1 structures. It is firmly anchored by hydrogen bonds to an aspartate (Asp155) and serine (Ser119), where the latter is located on the β4/β5-loop that influences substrate specificity.^5^ In PPEP-3, the equivalent residue Tyr161 points toward the center of the active-site cleft in all structures reported here (Figure 2B). In PPEP-2, the equivalent residue is an arginine (Arg145; Figure 2C), which forms a salt bridge with Glu113, which is also located on the β4/β5-loop. Furthermore, the S2′ pocket residue Leu179 in PPEP-1 is substituted by a more sterically demanding phenylalanine in PPEP-3 (Phe191), and by a valine (Val175) in PPEP-2 (Figures 2C and S3).

Another key difference in the active site is the conformation of the oxyanion hole residue Tyr190 in PPEP-3 (Tyr178 in PPEP-1 and Tyr174 in PPEP-2). In the substrate-unbound PPEP-3 structures of the wild type and the inactive double mutant E154A/Y190F, the corresponding side chain is rotated out of the active site into a catalytically incompetent conformation. In the PPEP-1 structure, it is in a catalytically productive conformation pointing toward the Zn^2+^ ion, while in the PPEP-2 structure, it distantly coordinates via its phenolic hydroxyl group the Cd^2+^ ion, which was introduced instead of the catalytic Zn^2+^ ion by the crystallization buffer. The reason for the particular conformation of this tyrosine in PPEP-3 is not clear, but it could be related to the presence of a bicine buffer molecule, which is coordinating the catalytic zinc ion in PPEP-3. This hypothesis is supported by the inhibiting effect of bicine on PPEP-3 activity (Figure S4).

The C-terminal domain of PPEP-3 is formed by the six helices η6, η7, and α5–α9 which carry the third zinc ligand, Glu197 on α7, and the aforementioned catalytically important Tyr190 on helix α6.

### Substrate binding induces large conformational changes in PPEP-3

**Figure 3:**
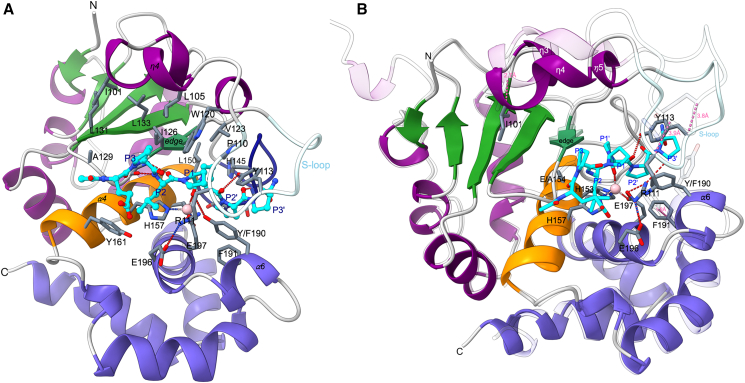
PPEP-3 substrate complex structure and induced changes (A) The substrate peptide is depicted in cyan color with the residues named according to Schechter & Berger. Residue 190 is a phenylalanine in the mutant crystallized with the substrate peptide. For this figure, it has been changed back *in silico* to tyrosine to visualize the interaction of the wild-type residue with the substrate/transition state. Hydrogen bonds are shown as red dotted lines. Depicted are all the residues that contact the substrate and/or line the specificity pockets. (B) Overlay of the substrate-bound and non-bound (light colors, transparent) structures. The major movements caused by substrate binding are indicated by pink distances, which are 2.7 Å for Ile101, 1.8 Å for Phe191, and 3.8 for Tyr113. Residue Phe190 is the actual amino acid in the crystal structure with its experimentally determined conformation.

To determine the structure of the PPEP-3-substrate complex, a proteolytically inactive double mutant E154A/Y190F was prepared. Because its residual activity of less than 1% prevented the crystallization of a substrate complex, an attempt was made to deplete the catalytic zinc ion by EDTA and/or ortho-phenanthroline treatment. This was partially successful, reducing the Zn^2+^ occupancy to about 40%–50% as judged by crystallographic refinement. In contrast, in PPEP-1, the zinc ion was virtually completely removed after such treatment.^11^ Still, this procedure led to the successful structure determination of the substrate complex of PPEP-3. In two protomers, the entire substrate chain is well resolved (Figure S10), while in the other two, the N-terminal two residues have weak density. Substrate binding occurs roughly antiparallel to the edge strand β4 (Figure 3A).

In the PPEP-1 cocrystal structures, substrate binding causes the closure of the S-loop via Trp103 and Lys101. Trp103 forms a hydrogen bond with the carbonyl oxygen of the P2′ residue. Meanwhile, Lys101 establishes hydrogen bonds with the glutamate tandem Glu184,185 (which includes the zinc-coordinating glutamate residue) as well as with the side chain of asparagine at the P2 position. S-loop closure proceeds similarly in the complex of PPEP-3 with the Ac-EPLPPPP-NH_2_ peptide, where the S-loop has moved by about 4 Å to close over the occupied active-site cleft (Figure 3B) and hydrogen bonds via Tyr113 to the P2′ carbonyl oxygen. PPEP-1’s Lys101 is replaced in PPEP-3 by Arg111, which also hydrogen bonds with the γ-carboxylate groups of Glu196 and the zinc-coordinating Glu197 (Figure 3). In contrast to the preferred asparagine at the P2 position in the PPEP-1 cocrystal, no hydrogen bonds can be formed between PPEP-3’s Arg111 and the leucine at the P2 position.

Additional conformational changes are observed for PPEP-3 (Figure 3B). Compared to PPEP-1, more movement (∼3 Å) is observed at the η3/η4 loop. This brings Ile101 of the η3/η4 loop in closer proximity to the aliphatic residues Leu131 and Leu133 on the β5 strand located directly beneath, thereby increasing the van der Waals interactions between these elements and possibly aiding in the closure of the neighboring S-loop.

Another substrate binding-induced change is observed in PPEP-3 but not in PPEP-1 at the α6 helix, which moves about 2 Å away from the active site, thereby creating space for the P2′ residue. This α6 helix bears the oxyanion-forming Tyr190, which is rotated outwards in the unbound structure, thus unable to stabilize a tetrahedral transition state in this conformation. In the substrate-bound conformation, the substituted Phe190 residue, which was introduced instead of the tyrosine to prevent substrate cleavage, rotates toward the catalytic zinc ion. This shift in the α6 helix positions the aromatic side chain similarly to the equivalent Tyr178 in PPEP-1’s apo-structure (PDB: 5A0P and 5N12), thus enabling catalytic activity.

The neighboring Phe191 is part of the S2′ pocket and restricts the size of P2′ residues in the substrate (Figure 3). The movement of Phe191 by nearly 2 Å increases the S2′ pocket’s size and thereby accommodates the presence of a proline residue at the P2′ position (Figure 3B). However, the large phenylalanine side chain may still interfere with sterically more demanding residues at the P2′ position, e.g., the valine of the PPEP-1 substrate, as is further discussed below.

### Substrate recognition

**Figure 4:**
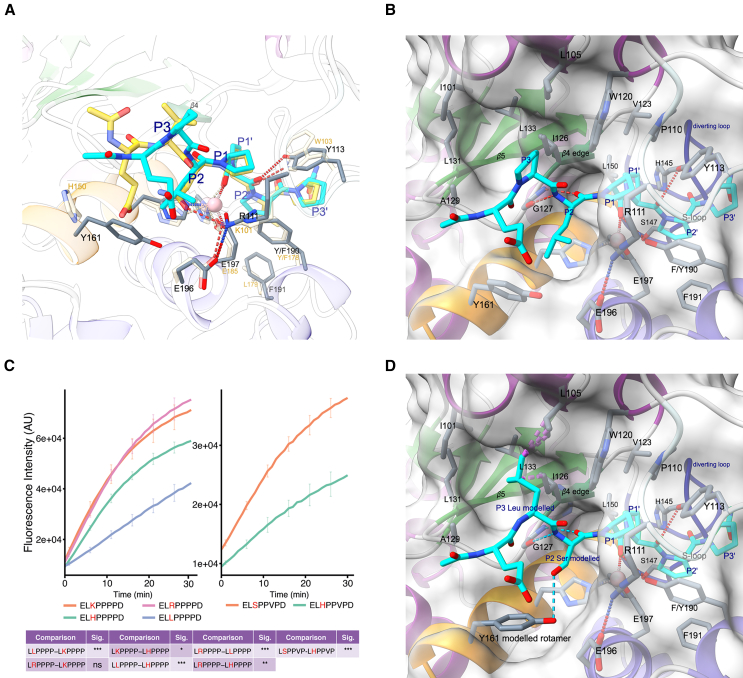
PPEP-3 substrate recognition (A) Overlay of the PPEP-3 (substrate colored in cyan as before but cartoon transparent, black residue labels) with PPEP-1 substrate complex (white cartoon, substrate shown as yellow sticks, golden labels). Hydrogen bonds are indicated by red dashed lines. (B) PPEP-3 substrate complex with molecular surface emphasizing the S3 and S2 substrate pockets. (C) Time-course of PPEP-3 mediated cleavage of FRET-quenched peptides with the sequence Lys(Dabcyl)-EL(R/H/K/L)PPPPD-Glu(EDANS) (left graph) and time-course of PPEP-3 mediated cleavage of FRET-quenched peptides with the sequence Lys(Dabcyl)-EL(S/H)PPVPD-Glu(EDANS) (right graph). The curves represent the mean and standard deviation (SD) of three replicates. Differences in the cleavage efficiency of the peptides were determined by statistical analyses of the baseline-corrected areas under the curve, which were a one-way ANOVA + Tukey (HSD) and an unpaired *t* test for the left and right graphs, respectively. *p* > 0.05 (ns), *p* < 0.05 (∗), *p* < 0.01 (∗∗), *p* < 0.001 (∗∗∗). (D) *In silico* modeling of the preferred leucine and serine residues in P3 and P2 positions, respectively. Clashes are indicated by purple broken lines, hydrogen bonds by cyan lined for modeled residues or red dashed lines for others.

The specificity of PPEPs to hydrolyze Pro-Pro peptide bonds originates most likely from the interactions between the P1-P1′ prolines and the S1 and S1′ pockets, as well as from the shape of the active site, which is at the substrate’s C-terminal end sculpted by the diverting loop and fitting to the substrate’s main solution conformer.^11^ The PPEP-3 and PPEP-1 substrates adopt a very similar, double-kinked conformation (Figure 4A). The interactions of the P1 and P1′ prolines in PPEP-3 are similar to those in PPEP-1, and most of these residues are conserved in the other PPEPs (Figures S3 and S5). The P1 proline residue is enclosed in the hydrophobic S1 pocket formed by Pro110, Trp120, and Tyr113. In addition, its main chain carbonyl oxygen coordinates the catalytic zinc ion. The P1′ proline side chain interacts with Val123, Leu150, Tyr113, His145, and the zinc-coordinating His153. Most notable is the hydrogen bonding of the main chain carbonyl oxygen of the P1′ proline with the phenolic hydroxyl group of Tyr113, which is located on the S-loop (Figures 4A, 4B, and S5). Of all the residues involved in these protease-substrate interactions, only this Tyr113 residue is different in PPEP-1, where it is a tryptophan (Trp103) (Figures 4A and S3). These residues are implied in S-loop closure and are important for catalysis. Interestingly, substitution of Trp103 in PPEP-1 by a tyrosine dropped the activity to about 7% of the wild type.^11^ It is unclear why this is not the case in PPEP-3 and related PPEPs with a tyrosine at this position.^5^

### The importance of Tyr161 for the P2 specificity of PPEP-3

The P2 specificity of PPEP-3 is characterized by a preference for the basic residues histidine, arginine, and lysine (Figure 1). However, these residues are overrepresented in the EICs and logo due to their efficient ionization in LC-MS/MS.^7^^,^^15^ Still, cleavage assays using FRET-quenched peptides showed a preference for the basic residues over leucine at the P2 position (Figure 4C), which was used to produce the cocrystal and is also observed in the logo in Figure 1. In the crystal structure, this P2 leucine residue interacts with PPEP-3 through both main chain hydrogen bonds with Gly127 on the edge strand and van der Waals interactions by the side chain (Figures 3 and 4B). The leucine side chain is snugly embedded in the S2 pocket, which is formed by the residues Arg111, Gly127, Gly128, His157, Tyr161, Glu196, and Glu197.

Following the basic residues, serine is the next most abundant residue observed at the P2 position (Figure 1). Comparison of FRET-quenched peptides with either serine or histidine at the P2 position shows a preference for the former (Figure 4C). This can be explained by the hydrogen bonding with Tyr161 observed after modeling the serine at the P2 (Figure 4D). However, this hydrogen bond is only present when Tyr161 adopts the rotamer observed in the apo structure. A leucine at the P2 position necessitates a larger S2 pocket, which causes the Tyr161 side chain to move away from the active site in the protease-substrate complex by mainly adopting a different χ^2^ dihedral angle (Figure 4D).

An increase of the S2 pocket size by side chain conformations of Tyr161 that differ from the apo crystal structure is also needed to explain the presence of the basic residues at the P2 position in the logo (Figure 1). Substitution of the P2 leucine for histidine, arginine, and lysine reveals steric clashes with Tyr161 in some rotamers, but for example arginine can be modeled with only minor clashes with the side chain of the preceding glutamate in the Ac-EP(L/R)PPPP-NH_2_ peptide (Figure S6). Also, possibly Tyr161 could adopt a similar conformation as His150 in PPEP-1 (Figure 4A), thereby allowing the larger basic residues to fit the S2 pocket.

### The P3 specificity of PPEP-3 is owed to a large and hydrophobic S3 pocket

PPEP-3 mostly tolerates hydrophobic residues at the P3 position, although also histidine, glycine, and glutamine are observed (Figure 1). The S3 pocket consists of His104, Leu105, and Trp120 and is backed up by Ile126 (Figures 4B and 4D). The many hydrophobic residues in this pocket, together with the location at the surface of the protein, explain the preference for hydrophobic residues due to both hydrophobic and van der Waals interactions at the P3 position. For example, leucine is the preferred residue at the P3 position, a preference that can be explained by its hydrophobic character. Nevertheless, a straightforward exchange of the P3 proline side chain by the one of leucine leads to some clashes (Figure 4D). These can be remedied by slight adjustments of the substrate’s main chain conformation, mainly by altering the *ϕ* angle of the P3 residue from −73° to −105°, with the latter not being accessible for proline.

When comparing the P3 specificity between PPEP-3 and other PPEPs, the high occurrence of the phenylalanine residue in the logo stands out (Figure 1). Residues Leu105 and Trp120 of PPEP-3’s S3 pocket are well conserved in PPEP-2, -3, and -4. In addition, Ile126, which closes the S3 pocket, is replaced by either leucine (PPEP-1 and PPEP-4) or valine (PPEP-3), which are residues with similar physicochemical properties. The main difference is His104 in PPEP-3, which is replaced by a tyrosine in the other PPEPs (Figure S3). In the PPEP-1 cocrystal, the hydroxyl group of the Tyr94 residue (PDB: 6R5C) restricts the size of the hydrophobic P3 pocket compared to the His104 in PPEP-3. *In silico* replacement of His104 in PPEP-3 by a tyrosine restricts the S3 pocket such that it cannot accommodate the bulky phenylalanine side chain anymore at the P3 position without major deviations in the substrate backbone (Figures 4D and S7).

### PPEP-3 displays a strong preference for prolines at the P2′ and P3′ positions

The P2′ residue is deeply buried in the tunnel created by S-loop closure, while the P3′ residue is surfacing out. In the PPEP-1 and PPEP-3 substrate complex structures, the P2′ residue hydrogen bonds to the amide NH group of the backbone of a residue on the diverting loop, which is Gly146 in PPEP-3 and Asp135 in PPEP-1.

**Figure 5:**
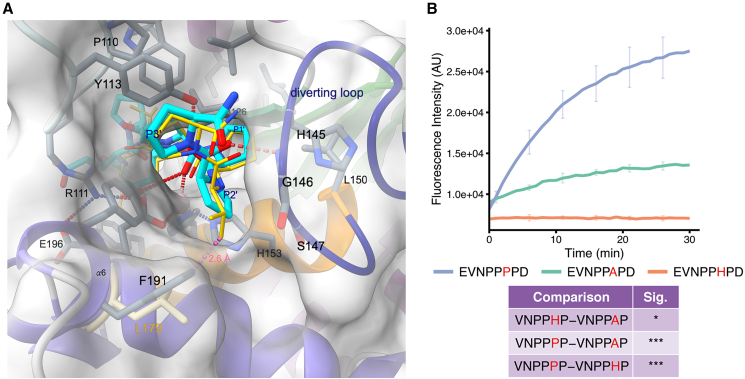
Structural analyses of the prime-side specificity of PPEP-3 (A) A view along the prime-side residues in the active center. Shown is PPEP-3 in the colors used before. The PPEP-1 complex structure (PDB: 5A0X) is overlaid, but only the substrate peptide (yellow sticks) and Leu179 are shown. Hydrogen bonds are indicated by red and clashes by purple dashed lines. (B) Time-course of PPEP-3 mediated cleavage of FRET-quenched peptides with the sequence Lys(Dabcyl)-EVNPP(P/A/H)PD-Glu(EDANS). The curves represent the mean and SD of three replicates. Differences in the cleavage efficiency of the peptides were determined by statistical analysis of the baseline-corrected areas under the curve by a one-way ANOVA + Tukey (HSD). *p* > 0.05 (ns), *p* < 0.05 (∗), *p* < 0.01 (∗∗), *p* < 0.001 (∗∗∗).

The results from the combinatorial peptide library are in good agreement with previous data on the prime-side specificity of PPEP-3, which showed a high preference for proline residues at the P2′ and P3′ positions.^7^ The small differences observed between the logo in Figure 1 and the previously reported logo^7^ are mainly due to altered inclusion criteria of product peptides. The preference for a proline at the P3′ position is a shared characteristic of PPEPs.^6^^,^^7^ In PPEP-1, Trp103 interacts with the pyrrolidine ring of the proline at the P3′ position in a parallel aliphatic-aromatic stacking interaction and forms a hydrogen bond to the carbonyl oxygen of the P1′ proline.^11^ In PPEP-3, the corresponding residue is Tyr113, which interacts with the P3′ proline similar to Trp103 in PPEP-1, i.e., through an aliphatic-aromatic CH/π interaction (Figure 5A). In addition, the P3′ proline residue is oriented at a 90° angle to Phe190, which was introduced for the oxyanion hole tyrosine to create the proteolytically inactive PPEP-3 (Figure 4C). The partially positive carbon of the pyrrolidine ring (Cδ) interacts with the negative electrostatic potential of the aromatic ring of Phe190 (Tyr190 in the wild type) in a second CH/π interaction.^16^

While all characterized PPEPs tolerate a proline at the P2′ position, PPEP-3 displays the strongest preference for this residue (Figure 1^6^^,^^7^). Notable differences are observed when comparing the S2′ pocket of PPEP-3 to that of PPEP-1 (Figure 5A). The most significant difference impacting P2′ specificity is the presence of a sterically demanding phenylalanine (Phe191) in the S2′ pocket of PPEP-3. In PPEP-1, this residue is a leucine (Leu179) and in PPEP-2 a valine (Val175), both are considerably smaller. *In silico* substitution of the P2′ proline with the Cβ-branched valine in the PPEP-3 complex structure results in a steric clash (Figure 5A). This clash is not too severe, though, and PPEP-3 still cleaves peptides with a P2′ valine (Figure 4C), but it is the major reason for the preference of proline over valine at the P2′ position.

In addition to proline, the logo in Figure 1 shows the presence of alanine and histidine at the P2′ position. A modeled substitution of the P2′ proline by alanine does not cause a steric clash but reduces the amount of van der Waals interactions between the substrate residue and Phe191 (Figure 4C). In addition, proline residues increase backbone rigidity owing to the restricted *ϕ* angle compared to other residues, thereby reducing the entropy loss of the substrate upon binding. A substrate with an alanine at the P2′ loses more entropy to adopt the right conformation to fit the active site.

The presence of histidine at the P2′ position in the logo (Figure 1) is surprising due to its size, and this residue produces steric clashes when trying to model its side chain in between the Phe191 and P3′ proline. The signal for the PHP (P1′-P3′) product peptide is low compared to PPP and PAP (P1′-P3′) peptides, and histidine residues are overrepresented due to the efficient ionization of histidine-containing peptides.^7^^,^^15^ Indeed, an assay using FRET-quenched peptides showed a preference for a proline at the P2′ position, a lower activity for alanine, and no activity when a histidine occupied the P2′ position (Figure 5B).

## Discussion

Using synthetic combinatorial peptide libraries in combination with LC/MS/MS, we comprehensively characterized the substrate specificity of PPEP-3. These results, integrated with the structural data presented here, offer mechanistic insights into substrate recognition by PPEP-3. Our findings, along with previous data, reveal a strong preference for proline residues at the prime-side substrate positions. This specificity is likely due to both steric constraints within the substrate-binding pockets and favorable interactions between prolines at the P2′ and P3′ positions and the peptidase. Additionally, the preference for proline at the P2′ position may stem from the increased rigidity of the peptide backbone, which minimizes entropy loss upon substrate binding.

In contrast, the non-prime-side specificity of PPEP-3 has been less thoroughly investigated. Earlier FRET-based cleavage assays indicated that PPEP-3 tolerates sequences such as VNP, PLP, PSP, and, to a lesser extent, DNP at the P3-P1 positions, particularly in the context of prime-side prolines. Given that PPEP-2 and PPEP-4 accommodate a leucine at P2^6^, the peptide Ac-EPLPPPP-NH2 was selected for co-crystallization. Although co-crystallization was successful with Ac-EPLPPPP-NH2, current insights suggest that alternative peptides might exhibit higher binding efficiency. Nonetheless, use of Ac-EPLPPPP-NH2 revealed a conformational shift in the Tyr161 side chain, likely representing a general mechanism for expanding the S2 pocket to accommodate bulkier residues such as histidine, arginine, or lysine at P2.

Although PLPPPP (P3-P3′) is cleaved in assays using FRET-quenched peptides,^7^ we did not identify the PTEDAVPLP product peptide in our combinatorial peptide library experiment. Previously, we showed that in the context of PLPPPP substituting the P2′ proline for a valine residue eliminates PPEP-3 activity.^7^ However, the FRET-quenched peptides containing LSPPVP and LHPPVP (P3-P3′) were cleaved by PPEP-3 (Figure 4C). This indicates that valine is only tolerated at the P2′ position when the non-prime-side residues are highly favored by PPEP-3. Since the less favored motif PLP (P3-P1) is most likely only tolerated in the context of PPP and possibly PAP (P1′-P3′), the resulting PTEDAVPLP product peptides in our combinatorial peptide library assay may not exceed the limit of detection. This phenomenon is especially observed for PPEP-3. For the other PPEPs that display a more variable prime-side specificity, more peptides displaying a specific non-prime-side sequence are cleaved, which increases the non-prime-side product peptide signals in our LC-MS/MS analyses.

In PPEP-1, Trp103 is essential for activity due to its π-CH interactions with the P3′ proline and the hydrogen bonding of the side chain nitrogen with the carbonyl oxygen of the P1′ residue in the protease-substrate complex.^11^ Mutation of this residue to alanine, histidine, phenylalanine, and tyrosine greatly diminished PPEP-1 activity.^11^ In PPEP-3, the corresponding residue Tyr113 interacts similarly with the proline residue at P3′ but also produces a similar hydrogen bond with the P1′ carbonyl oxygen.

Based on the preference of PPEP-3 for all prolines at the prime-side, we searched for secreted proteins possessing four consecutive prolines (P↓PPP, P1-P3′) in the G*. thermodenitrificans* proteome.^7^ This search identified two proteins with either PSP↓PPP or DNP↓PPP as the putative PPEP-3 cleavage site, with PSP↓PPP being the far better substrate.^7^ However, strong binding between PPEP-3 and the non-prime-side residues allows for more flexibility at the P2′ position (Figure 5B). Based on our new combinatorial peptide library results, we performed a search for endogenous substrates that included proteins that contained the motif (L/F)(H/R/K/S)P↓P (P3-P1′), resulting in the identification of 50 proteins. Of these 50 proteins, only a single protein, GTNG_0399, was predicted to possess a signal peptide for secretion by SignalP 6.0 (https://services.healthtech.dtu.dk/services/SignalP-6.0/). This candidate substrate is a spore coat N-acetylmuramic acid deacetylase containing an LRPPRG site. Given the peptide library results shown in Figure 1, the combination of an arginine at the P2′ and a glycine at the P3′ is most likely not tolerated by PPEP-3. In addition, our LC-MS/MS analysis does not indicate the presence of a PRGGGLEEF product peptide. Therefore, the protein GTNG_0956 containing the putative cleavage site PSP↓PPP (P3-P3′)^7^ remains the most likely endogenous candidate, especially since we can explain the preference for a serine residue at the P2 position due to the hydrogen bonding with Tyr161 (Figure 4D). Alternatively, the biological PPEP-3 substrate could also originate from a different organism.

The unique ability to specifically hydrolyze Pro-Pro bonds could be advantageous in applications that necessitate precise proteolysis, such as the removal of affinity tags.^17^ In addition, several industrial processes, e.g., the breakdown of collagen for meat tenderization, require proteolysis of proline-rich proteins.^18^^,^^19^^,^^20^ Although PPEP specificity is too strict to degrade a variety of proteins, directed mutagenesis could render these proteases more promiscuous while retaining the Pro-Pro specificity. A detailed understanding of the factors that determine PPEP specificity can aid in the development of PPEPs suitable for industrial applications. In this study, we shed more light on the structure-function relationship of PPEPs by combining an experimentally determined protease-substrate complex with an in-depth substrate specificity profile. This combination of techniques can be a valuable tool to study the mechanisms governing substrate specificity in other PPEPs or, with some adaptations to the peptide libraries, other proteases.

### Limitations of the study

The synthetic combinatorial peptide libraries are produced using the mix-and-split (or split-and-pool) method, which is a stochastic process. The peptides are synthesized on 1.000.000 beads, while 130.321 possible combinations are possible in our peptide design. Due to the stochastic nature of the peptide synthesis, it is possible not all peptides are present in the peptide library. Additionally, the intensity of the substrate peptide fragments that are used for quantification is influenced by their behavior in LC-MS/MS. E.g., peptides containing the basic residues arginine, lysine, and histidine are more often ionized than others, which leads to the overrepresentation of these residues in the logos displaying the substrate preference of PPEP-3.

## Resource availability

### Lead contact

Requests for further information and resources should be directed to and will be fulfilled by the lead contact, Ulrich Baumann (ubaumann@uni-koeln.de).

### Materials availability

This study did not generate new unique reagents.

### Data and code availability

• This paper does not report original code. The mass spectrometry proteomics data have been deposited to the ProteomeXchange Consortium via the PRIDE^21^ partner repository with the dataset identifier PXD061585. Coordinates and structure factors have been deposited with the PDB under the IDs 9G0J, 9G3T, and 9G5J.
• Any additional information required to reanalyze the data reported in this paper is available from the lead contact upon request.

## Acknowledgments

The work was funded by an 10.13039/501100013427ENW-M grant (OCENW.KLEIN.103) from the 10.13039/501100024871Dutch Research Council (NWO). Crystals were grown at the Cologne Crystallization facility (C_2_f), which is supported by 10.13039/501100001659DFG grant INST 216/949-1 FUGG. We would like to thank the staff of the ESRF and EMBL Grenoble for assistance and support in using beamlines ID30A-3 and ID30B under proposal number MX2485 and MX2603. The support from the 10.13039/501100008001University of Cologne is acknowledged.

## Author contributions

P.H. and U.B., conceptualization, supervision, and investigation; B.C., F.W., and U.B., formal analysis; P.H., funding acquisition; B.C., F.W., L.P., R.A.C., A.H.d.R., and J.C., investigation; H.C.v.L., supervision; B.C., F.W., P.H., and U.B., writing – original draft; B.C., F.W., P.H., U.B., and J.C., writing – review and editing.

## Declaration of interests

The authors declare no competing interests.

## STAR★Methods

### Key resources table

**Table:** 

REAGENT or RESOURCE	SOURCE	IDENTIFIER
**Bacterial and virus strains**		

*Escherichia coli* DH5α	Thermo Fisher Scientific	EC0112
*Escherichia coli* BL21 (DE3)	Fisher Scientific/Invitrogen	10328512

**Chemicals, peptides, and recombinant proteins**		

PPEP-3	This paper	N/A
PTEDAVXXPPXXEZZO non-prime-side combinatorial peptide library	Claushuis et al.^6^	N/A
JZEXXPPXXGGLEEF prime-side combinatorial peptide library	Claushuis et al.^7^	N/A
Ac-EPLPPPP-NH2	This paper	N/A
Lys(Dabcyl)-EXXPPXXD-Glu(Edans) (X positions are varied)	This paper	N/A
Restriction endonuclease NdeI	New England Biolabs	Catalog No R0111S
Restriction endonuclease XhoI	New England Biolabs	Catalog No R0146S
Restriction endonuclease DpnI	New England Biolabs	Catalog No R0176S
Isopropyl-β-D-thiogalactopyranoside (IPTG)	Biotrend	Catalog No NB-45-00111
Kanamycine sulfate	Carl Roth	Catalog No T832.3
DNAse I	AppliChem	
EDTA	Applichem	Catalog No 131669
*Ortho*-phenantroline	Sigma-Aldrich	Cat #P9375

**Critical commercial assays**		

Crystallization screen Morpheus	Molecular Dimensions	Cat # MD1-46

**Deposited data**		

Raw LC-MS/MS data	This paper	ProteomeXchange ID: PXD061585
PPEP-3 (apo) structure	This paper	PDB: 9G0J
PPEP-3 E154A/Y190F (apo) structure	This paper	PDB: 9G3T
PPEP-3 E154A/Y190F in complex with Ac-EPLPPPP-NH2	This paper	PDB: 9G5J

**Oligonucleotides**		

Primer forward: Glu154 to Ala point mutation: CTGCACGCATTCGCGCACTCTCTGG	This paper	N/A
Primer reverse: Glu154 to Ala point mutation: CGAATGCGTGCAGTTCCAGGTTG	This paper	N/A
Primer forward: Tyr190 to Phe point mutation: GAATACTTCTTCCTGACCTACCCGG	This paper	N/A
Primer reverse: Tyr190 to Phe point mutation: CAGGAAGAAGTATTCACGCGGGAAC	This paper	N/A

**Recombinant DNA**		

Plasmid: pET28a	Sigma-Aldrich	Cat# 69864
Plasmid: pET28a-PPEP-3	This paper	N/A
Plasmid: pET28a-PPEP-3 (E154A)	This paper	N/A
Plasmid: pET28a-PPEP-3 (E154A/Y190F)	This paper	N/A

**Software and algorithms**		

PyMOL version 2.5.5	Schrödinger	https://www.pymol.org/
USCF Chimera X	UCSF Resource for Biocomputing, Visualization, and Informatics	https://www.cgl.ucsf.edu/chimerax/
Rstudio version 2024.12.1	Posit Software PBC	https://posit.co/download/rstudio-desktop/
R version 4.4.2	The R Project for Statistical Computing	https://www.r-project.org/
XDS version Jun 30, 2024 BUILT = 20241002	Kabsch et al.^22^	https://xds.mr.mpg.de/
PHENIX version 1.21.2_5419	Adams et al.^23^	https://phenix-online.org/documentation/index.html
Coot version 0.9.8.95 and previous	Emsley et al.^24^	https://www2.mrc-lmb.cam.ac.uk/personal/pemsley/coot/
Proteome discoverer version 2.5.0.400	Thermo Fisher Scientific	https://www.thermofisher.com/nl/en/home/industrial/mass-spectrometry/liquid-chromatography-mass-spectrometry-lc-ms/lc-ms-software/multi-omics-data-analysis/proteome-discoverer-software.html?erpType=Global_E1
Mascot version 2.2.7	Matrix Science	www.matrixscience.com
Skyline version 23.1.0.268	MacCoss Lab Software	https://skyline.ms/project/home/begin.view

**Other**		

Cell disruptor	I&L Biosystems	N/A
Ni-NTA superflow resin	Qiagen	Cat# 30410
HiLoad Superdex 200 16/600 column	Cytiva	Cat # 28989335
EnVision 2105 Multimode Plate Reader	Perkin Elmer	N/A
Pierce™ Monomeric Avidin Agarose	Thermo Fisher Scientific	Cat# 20228
Pierce High-Capacity Streptavidin Agarose beads	Thermo Fisher Scientific	Cat# 20361
Oasis HLB 1 cm3 30 mg reversed-phase solid-phase extraction cartridges	Waters	SKU: WAT094225
TentaGel S AC	Rapp Polymere	S30011.1G
Syro II peptide synthesizer	Multisyntech	N/A

### Experimental model and study participant details

#### Experimental source materials

The PPEP-3 protein used in this study originates from *Geobacillus thermodenitrificans* strain NG80-2 (gene: GTNG_1672). Protein expression was performed in *Escherichia coli* strain BL21 (DE3). *E. coli* BL21 (DE3) was grown in LB medium supplemented with 50 μg/mL kanamycin at 37 °C. Protein expression was induced using 0.5 mM Isopropyl 1-thio-Beta-D-galactopyranoside (IPTG) and expression was performed at 20 °C.

### Method details

#### Generation of constructs

The truncated version (amino acids 27–235, lacking the N-terminal predicted signal peptide) of the PPEP-3 gene (GTNG_1672) from *Geobacillus thermodenitrificans* strain NG80-2, codon optimized for *Escherichia coli*, was obtained in a pET28a vector using the restriction sites NdeI/XhoI. An active site double mutant was generated via the one-step site-directed mutagenesis protocol.^25^ For the E154A mutant, the PCR was performed using pET28a-PPEP3 as template and oligonucleotides JGP614-GeoPPEP_E154A_f: 5′-CTGCACGCATTCGCGCACTCTCTGG-3′ as well as JGP613-GeoPPEP_E154A_r: 5′-CGAATGCGTGCAGTTCCAGGTTG-3’. For the construct pET28a-PPEP3 (E154A/Y190F), the construct pET28a-PPEP3 (E154A) was used as a template and the oligonucleotides JGP615-GeoPPEP_Y190F_f: 5′-GAATACTTCTTCCTGACCTACCCGG-3′ and JGP616-GeoPPEP_Y190F_r: 5′-CAGGAAGAAGTATTCACGCGGGAAC-3′ were used to introduce the second mutation. A reaction was performed using 16 cycles with 98 °C denaturation for 30 s, 65 °C annealing for 30 s and 72 °C elongation for 6 min followed by a 2 min final elongation step. Subsequently a DpnI digest was conducted using 1 U DpnI (NEB) at 37 °C for 1 h. 2 μL of the reaction were transformed into chemically competent *E. coli* DH5α cells (Thermo Fisher Scientific), plated on kanamycin LB-agar selection plates and incubated overnight at 37 °C. Isolated plasmids were sequenced to identify positive clones.

#### Expression of recombinant PPEP-3

The wild type and mutant vectors were transformed into *E. coli* BL21 (DE3) (Invitrogen), plated on kanamycin supplemented LB-agar selection plates and incubated overnight at 37 °C. A preculture grown overnight at 37 °C from a single colony was used to inoculate 1 L expression cultures (LB, 50 μg/mL kanamycin) to an optical density (OD_600_) of 0.1. After incubation at 37 °C and reaching an OD_600_ of 0.7 expression was induced with 0.5 mM Isopropyl 1-thio-Beta-D-galactopyranoside (IPTG, BIOTREND). Protein expression was performed at 20 °C overnight. Cells were harvested by centrifugation at 4,000 x g, 4 °C for 20 min. Cell pellets were washed with Tris-buffered saline (TBS) [pH 7.5]. Cells were pelleted again and stored at −80 °C until further use.

#### Purification of PPEP-3

The proteins were purified as previously described with minor adjustments.^7^ The cell pellet from 2 L of culture was resuspended in TBS buffer with a volume of 5 mL per g of cell pellet of 20 mM Tris [pH 7.5], 300 mM NaCl) supplemented with 10 μg/mL DNaseI (AppliChem). Cells were lysed by running the suspension two times through a Cell Disruptor (I&L Biosystems) at 2.5 kbar. Cellular debris was pelleted by centrifugation at 10,000 x g, 4 °C for 10 min. The supernatant was cleared by ultracentrifugation at 165,000 x g, 4 °C for 30 min. The supernatant was adjusted with 1 M imidazole (pH 7.5) to a final concentration of 10 mM and loaded onto 2 mL Ni-NTA superflow resin (Qiagen). After two wash steps with TBS supplemented with first 10 mM and then 30 mM imidazole of about 10–15 column volumes until a stable base line was reached again, the protein was eluted with TBS containing 250 mM imidazole. The protein was concentrated and applied on a HiLoad Superdex 200 16/600 column (Cytiva) equilibrated with TBS. Protein fractions were collected, concentrated, and stored at a concentration of about 16 mg/mL in 20 mM Tris-HCl,pH 7.5, with 5 mM imidazole, at −80 °C until further use. The total yield was more than 30 mg of pure protein from 2 L culture. Protein concentration was determined at 280 nm using the molar extinction coefficient of 27,390 M-1 cm-1 (wild type) and 25,900 M-1 cm-1 (double mutant), respectively.

#### Crystallization of PPEP-3

Single crystals of substrate-unbound wild type, double mutant E154A/Y190F in unbound and Ac-EPLPPPP-NH2 were obtained by broad screening using sitting drop vapor diffusion crystallization with drop sizes of 300 nL. Protein (381 μM, 10 mg/mL) was pipetted in ratios of 1:2, 1:1 and 2:1 (protein to precipitant) in commercially available crystallization screens (Hampton Research). For substrate complex formation, the catalytic Zn^2+^ ion was removed by dialyzing the protein solution against buffer containing about 6 mM EDTA and 6 mM *ortho*-phenanthroline in order to avoid proteolysis, which occurs even in the double mutant albeit slowly. Crystal formation was observed in conditions Morpheus C1, C5, C9, E9 and H9. Best diffracting crystals for all the structures described here (wildtype, unbound double mutant and double mutant in complex with substrate peptide) were obtained from Morpheus E9 containing 10% w/v PEG 20,000, 20% v/v PEG MME 550, 0.3 M diethyleneglycol, 0.3 M triethyleneglycol, 0.3 M tetraethyleneglycol, 0.3 M pentaethyleneglycol, 0.1 M bicine/Trizma base pH 8.5. Single crystals were cryoprotected in a mixture of a precipitant solution containing 50% sucrose and flash-frozen in liquid nitrogen.

#### Data collection and structure determination

High-resolution data for structure determination were collected at ESRF on the beamline ID30A-3 using an Eiger X 4M detector (Dectris) or at beamline ID30B with an Eiger2 X 9M detector (Dectris). Datasets were processed with XDS.^22^ The structure was solved using molecular replacement employing the PPEP-1 coordinates (PDB: 5A0P)^9^ as a search model. Phasing and refinement were performed using the PHENIX package^23^ and model building with Coot.^24^ Data collection and refinement statistics are shown in Table S1.

#### Combinatorial peptide library assays

The combinatorial peptide libraries were synthesized, and assays were performed as previously described.^7^ In short, approximately 10 nmol of precleaned (on avidin column) peptides was incubated with 200 ng PPEP-3 for 3 h at 37 °C in PBS. A nontreated control was included. After incubation, the samples were loaded onto an in-house constructed column consisting of a 200 μL pipet tip containing a filter and a packed column of 100 μL of Pierce High-Capacity Streptavidin Agarose beads (Thermo, the column was washed four times with 150 μL of PBS before use) to remove the biotinylated peptides. The flow-through and four additional washes with 125 μL H2O were collected. The product peptides were desalted using reversed-phase solid-phase extraction cartridges (Oasis HLB 1 cm3 10 mg, Waters) and eluted with 200 μL of 30% acetonitrile (v/v) in 0.1% formic acid. Samples were dried by vacuum concentration and stored at −20 °C until further use. For the peptide library assays in which the non-prime- and prime-side libraries were combined, approximately 5 nmol of each library was used (10 nmol in total).

#### LC-MS/MS analyses

PPEP-3 product peptides were analyzed as previously described^6^ by online C18 nano-HPLC MS/MS with a system consisting of an Easy nLC 1200 gradient HPLC system (Thermo, Bremen, Germany) and an Orbitrap Fusion LUMOS mass spectrometer (Thermo). Peptides were injected onto a homemade precolumn (100 μm × 15 mm; Reprosil-Pur C18-AQ 3 μm, Dr Maisch, Ammerbuch, Germany) and eluted via a homemade analytical nano-HPLC column (30 cm × 75 μm; Reprosil-Pur C18-AQ 1.9 μm). The gradient was run from 2% to 40% solvent B (20/80/0.1 water/acetonitrile/formic acid (FA) v/v) in 52 min. The nano-HPLC column was drawn to a tip of ∼5 μm and acted as the electrospray needle of the MS source. The LUMOS mass spectrometer was operated in data-dependent MS/MS mode for a cycle time of 3 s, with HCD collision energies at 20 V, 25 V, and 30 V and recording of the MS2 spectrum in the orbitrap, with a quadrupole isolation width of 1.2 m/z. In the master scan (MS1) the resolution was 120,000, the scan range 350–1600, at an AGC target of 400,000 at a maximum fill time of 50 ms. A lock mass correction on the background ion m/z = 445.12003 was used. Precursors were dynamically excluded after *n* = 1 with an exclusion duration of 10 s and with a precursor range of 10 ppm. Charge states 1–5 were included. For MS2 the first mass was set to 110 Da, and the MS2 scan resolution was 30,000 at an AGC target of 100% @maximum fill time of 60 ms. The mass spectrometry data have been deposited to the ProteomeXchange Consortium via the PRIDE^21^ partner repository with the dataset identifier PXD061585.

#### LC-MS/MS data analysis

The LC-MS/MS data were analyzed as previously described.^6^ For the identification of product peptides after analysis of the mixed non-prime- and prime-side libraries, a database was generated containing all possible 9-mer product peptides that can be expected based on Pro-Pro cleavage (i.e., PTEDAVXXP and PXXGGLEEF).

Raw data were converted to peak lists using Proteome Discoverer version 2.5.0.400 (Thermo Fisher Scientific) and submitted to the in-house created databases using Mascot v. 2.2.7 (www.matrixscience.com) for peptide identification, using the Fixed Value PSM Validator. Mascot searches were with 5 ppm and 0.02 Da deviation for precursor and fragment mass, respectively, and no enzyme specificity was selected. Biotin on the protein N-terminus was set as a variable modification.

The database search results were filtered for product peptides that contained either PTEDAV or GGLEEF, were 9 residues in length, and contained no biotin. The resulting peptide lists were transported to Microsoft Excel, where duplicate masses and corresponding abundances were removed (e.g., the abundances of isomers PLPGGLEEF and PIPGGLEEF are listed twice, while this abundance is the total abundance of the two). The most abundant product peptides that together accounted for >90% of the total abundance were selected for further analysis. Further analysis was performed in Skyline 23.1.0.268 by importing the product peptides as FASTA along with the raw data files.^26^ The Extracted Ion Chromatograms (EICs) displaying the product peptides were created by plotting the intensities of the signals corresponding to the monoisotopic m/z values of both 1+ and 2+ charged peptides with a mass tolerance of 5 ppm.

#### FRET-quenched peptide synthesis

Peptides were synthesized by standard Fmoc-based solid-phase peptide synthesis (SPPS) on Tentagel S-Ac resin (Rapp Polymere, Tübingen, Germany) using a Syro II peptide synthesizer (MultiSyntech, Witten, Germany). Fmoc-protected amino acids carrying acid-labile side-chain protecting groups were coupled in N-methylpyrrolidone (NMP) using PyBOP/NMM with a 6-fold excess of amino acid, and coupling times of 1 h. Fmoc deprotection was performed with 20% piperidine in NMP, and washings were carried out with NMP. After chain assembly, peptides were cleaved from the resin and side-chains were deprotected using trifluoroacetic acid (TFA) containing 5% water. Final products were purified by reverse-phase HPLC. The purity of the peptides was assessed using MALDI-ToF MS (Figure S8).

#### FRET-quenched peptide cleavage assays

FRET-quenched peptide cleavage assays with PPEP-3 were performed using peptides with a Lys(Dabcyl)-EXXPPXXD-Glu(Edans) (the X positions varied between peptides). Assays were performed in triplicate in 150 μL PBS containing 200 ng enzyme and 50 mM FRET peptide. Peptide cleavage was analyzed using an Envision 2105 Multimode Plate Reader at 37 °C. Fluorescence intensity was measured every minute for 30 min, with 10 flashes per measurement. The excitation and emission wavelengths were 350 nm and 510 nm, respectively. The exact cleavage site was subsequently determined by MALDI-ToF MS (Figure S9).

#### Bioinformatic analyses and data visualization

Structures were analyzed using PyMOL (The PyMOL Molecular Graphics System, Version 2.5.5 Schrödinger, LLC) and USCF ChimeraX.^27^ The results of the FRET-quenched peptide cleavage assays were visualized using Rstudio (version 2024.12.1 build 563, Posit Software PBC, Boston, MA) with R (version 4.4.2, R foundation for Statistical Computing, Vienna, Austria). The results are visualized using the means from triplicate assays for the curves while displaying the standard deviation every 5 min.

### Quantification and statistical analysis

All statistical analyses were performed in Rstudio v2024.12.1 (Posit Software PBC) with R v4.4.2 (The R Project for Statistical Computing). In all time-course kinetic assays using FRET-quenched peptides, the data represent the mean ± standard deviation (SD). To find differences between the cleavage efficiency of peptides, the area under the curve corrected for the baseline (T = 0) was used as a metric. Statistical tests to compare the areas under curve included independent two-sample Student’s *t* test (two-tailed) and one-way independent ANOVA with Tukey (HSD) post-hoc test. Statistical significance is indicated as follows: *p* > 0.05 (ns), *p* < 0.05 (∗), *p* < 0.01 (∗∗), *p* < 0.001 (∗∗∗).
