# Supplementary material for: Structural analyses and substrate profiling of PPEP-3 provide new insights into the molecular basis of Pro-Pro endopeptidase specificity
Source: iScience. 2025 Dec 8;29(1):114360. doi: 10.1016/j.isci.2025.114360 (PMC12828418; doi:10.1016/j.isci.2025.114360)
Supplement: Document S1. Figures S1–S10 and Table S1 [file mmc1.pdf]

iScience, Volume *29*

Supplemental information

Structural analyses and substrate profiling of PPEP-3 provide new insights into the molecular basis of Pro-Pro endopeptidase specificity

Bart Claushuis, Fabian Wojtalla, Lisa Papenhagen, Robert A. Cordfunke, Arnoud H. de Ru, Hans C. van Leeuwen, Jeroen Corver, Paul J. Hensbergen, and Ulrich Baumann

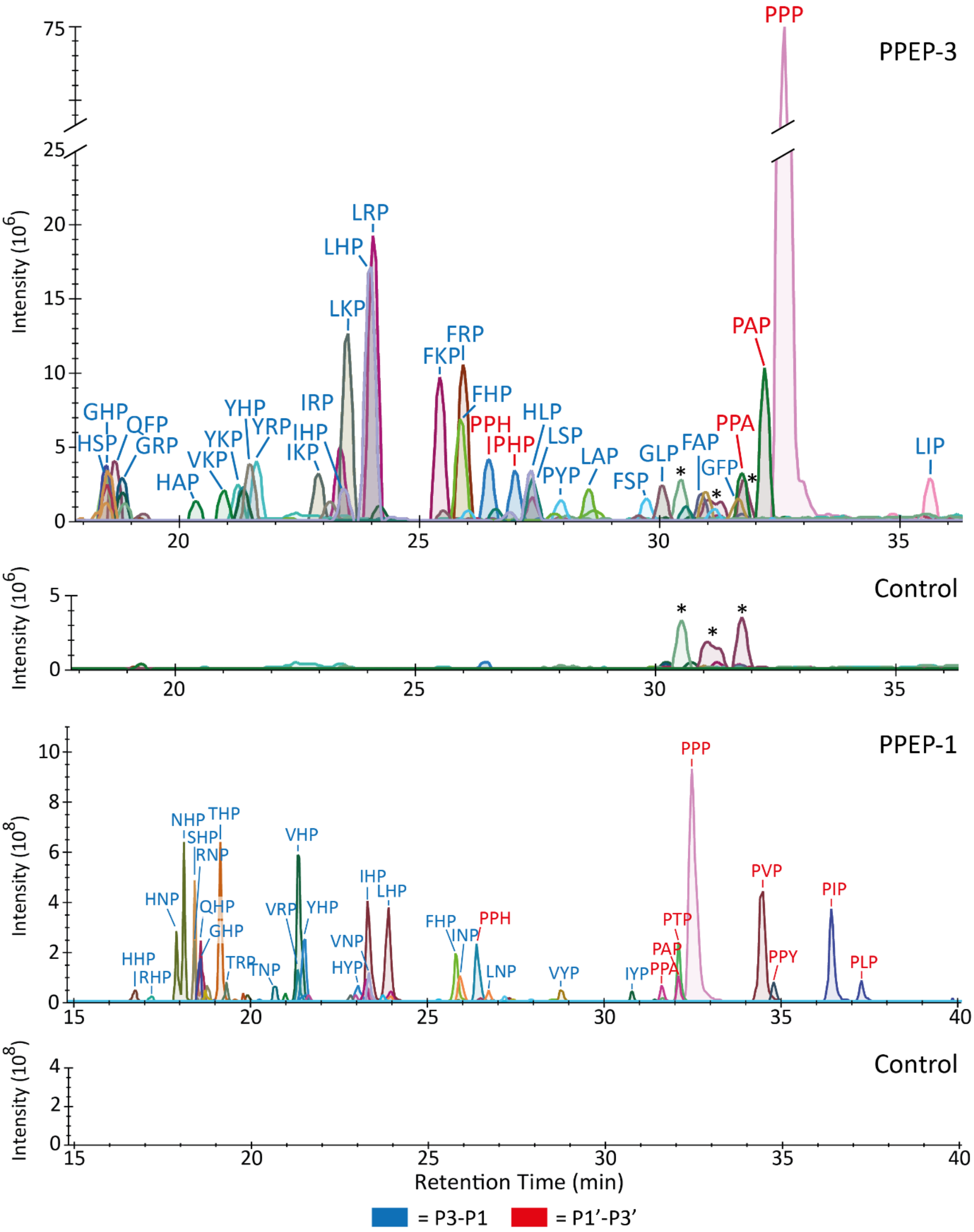

Supplementary Figure S1. PPEP-3 and PPEP-1 specificity for residues surrounding the Pro-Pro cleavage site. A comparison between the product peptides formed by PPEP-3 and PPEP-1 following incubation with the synthetic combinatorial peptide library. EICs were produced as described for Fig. 1 in the main text. The figure showing the results for PPEP-1 was adapted from Claushuis *et al.* [1], which is available under Creative Commons Attribution 4.0 International License.

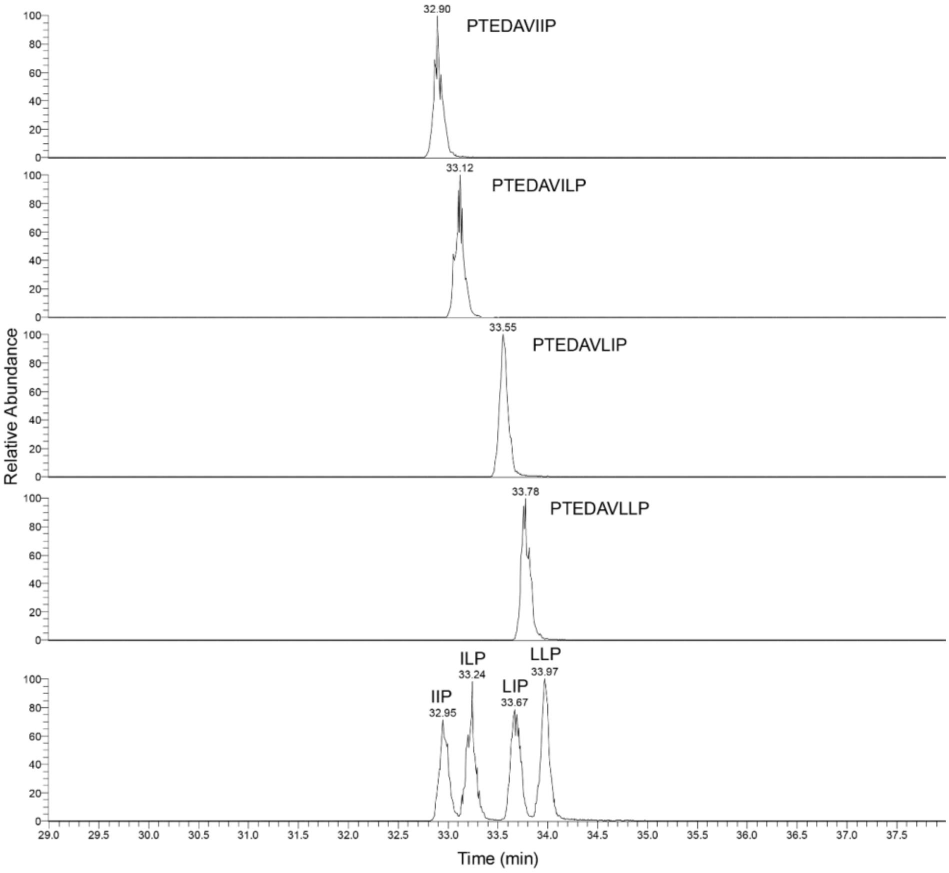

Supplementary Figure S2. Separation of the product peptides PTEDAVIIP, PTEDAVILP, PTEDAVLIP, and PTEDAVLLP. The retention times of synthetic peptides were analyzed on a C18 column using LC-MS/MS. EICs were produced by including m/z = 954.5142 ($[M + H]^+$) and m/z 477.7608 ($[M + 2H]^{2+}$) with a mass tolerance of 10 ppm.

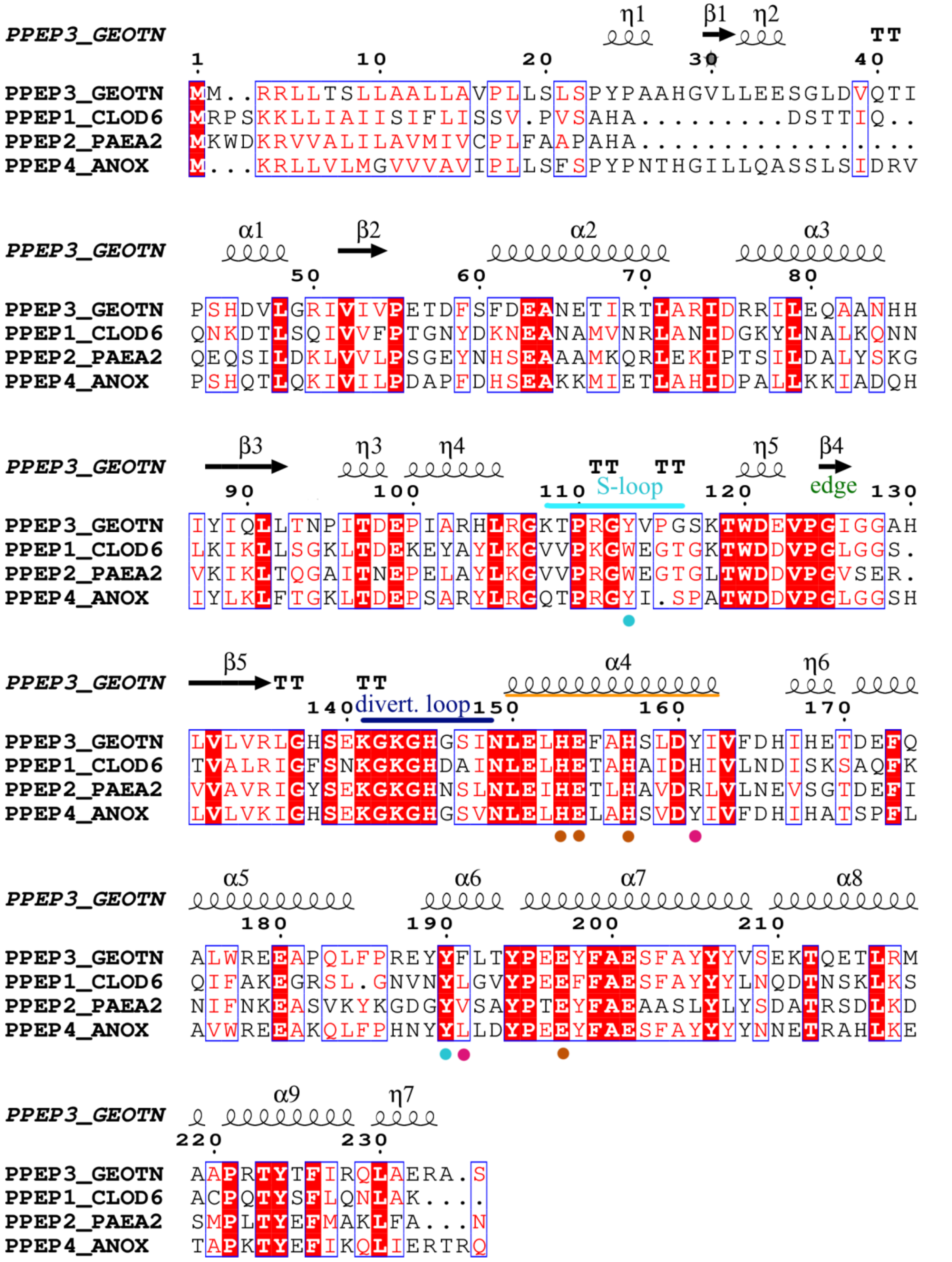

Supplementary Figure S3. Sequence alignment of PPEP-1, PPEP-2, PPEP-3, and PPEP-4. Uniprot identifiers are PPEP3_GEOTN: A4INY2; PPEP1_CLOD6: Q183R7; PPEP2_PAEA2: K4ZRC1; PPEP4_ANOX: A07W8IRZ3. "α" denotes regular alpha-helices, "β" are the beta-strands, η 3_{10} helices and "TT" are tight turns. Grey stars denote residues with alternate conformations. Figure was prepared with ESPRIPT[2].

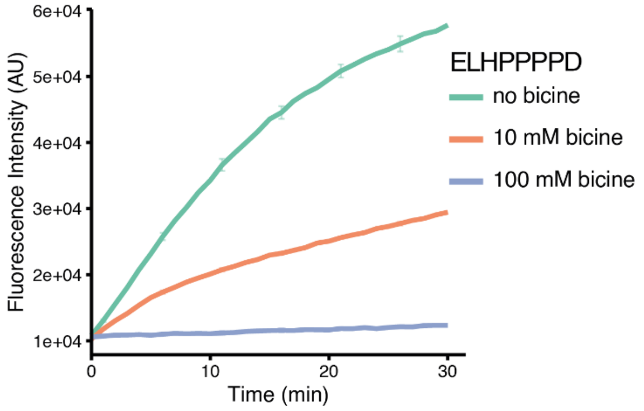

Comparison	Sig.
10 mM bicine vs. no bicine	***
100 mM bicine vs. no bicine	***
100 mM bicine vs. 10 mM bicine	***

Supplementary Figure S4. PPEP-3 activity is inhibited by bicine. Time-course of PPEP-3 mediated cleavage of FRET-quenched peptides with the sequence Lys(Dabcyl)-ELHPPPPD-Glu(EDANS). The curves represent the mean and standard deviation (SD) of three replicates. Differences in the cleavage efficiency of the peptides were determined by statistical analyses of the baseline-corrected areas under the curve by a one-way ANOVA + Tukey (HSD).

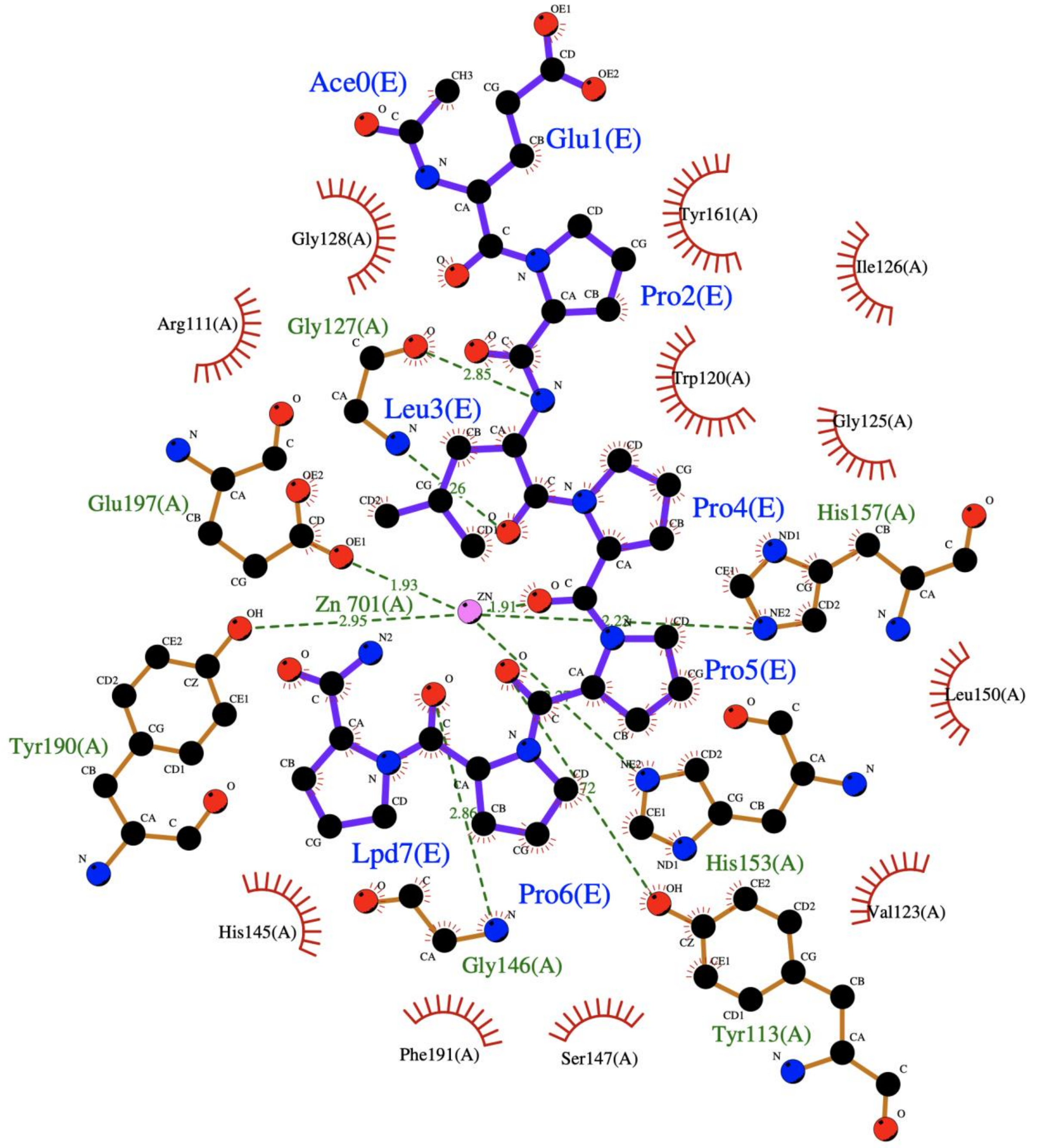

Supplementary Figure S5. Ligplot drawing of the substrate-protease interactions. Hydrogen bonds are indicated by green dashed lines, hydrophobic/van-der-Waals interactions by the curved lines with the spikes. The figure was prepared using Ligplot+[3].

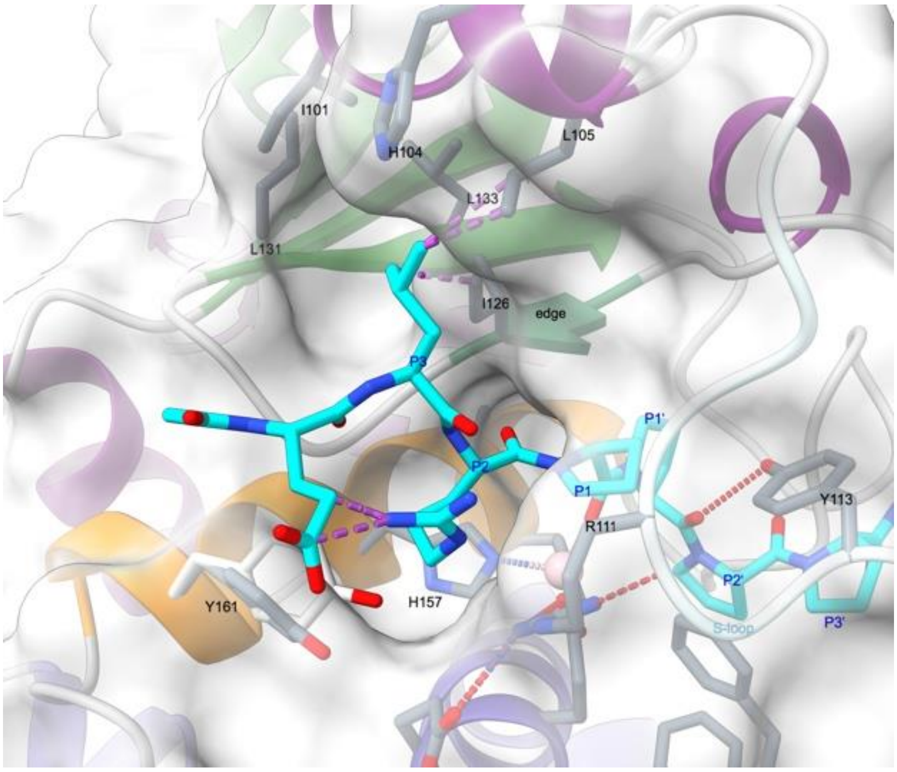

Supplementary Figure S6. Modeling of an arginine at P2 position. Rotation of Tyr161 (grey sticks in the peptide-bound conformation and white for the unbound crystal structure) is necessary to accommodate arginine in the S2 pocket. For this particular rotamer of arginine, clashes occur only with the side chain of the glutamic acid at the P4 position. These could be remedied by choosing another rotamer for this residue.

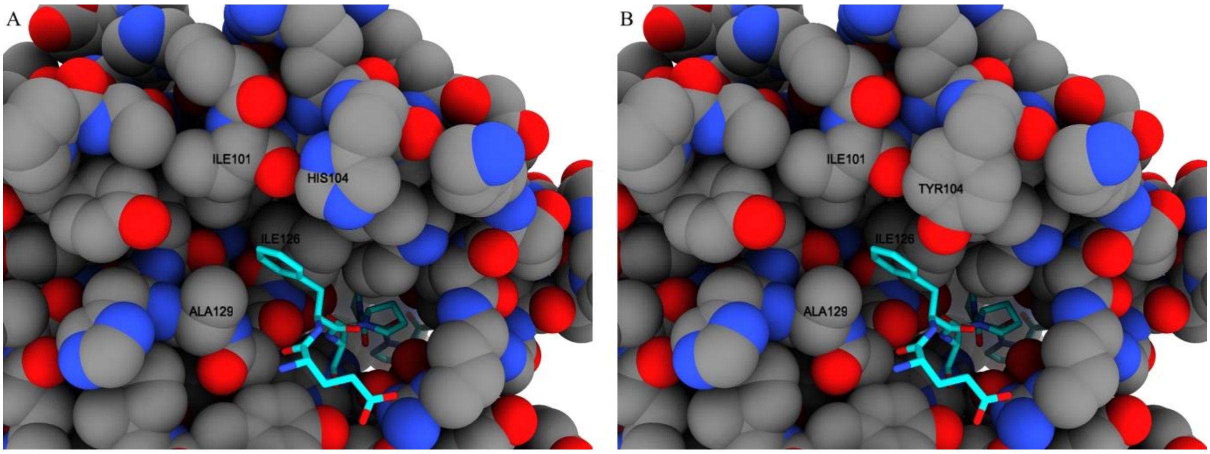

Supplementary Figure S7. The influence of His104 for the acceptance of a phenylalanine at P3. Substitution of the histidine (A) by a tyrosine (B) as in PPEP-1, -2, and PPEP-4 creates steric clashes with the bulky phenylalanine side chain.

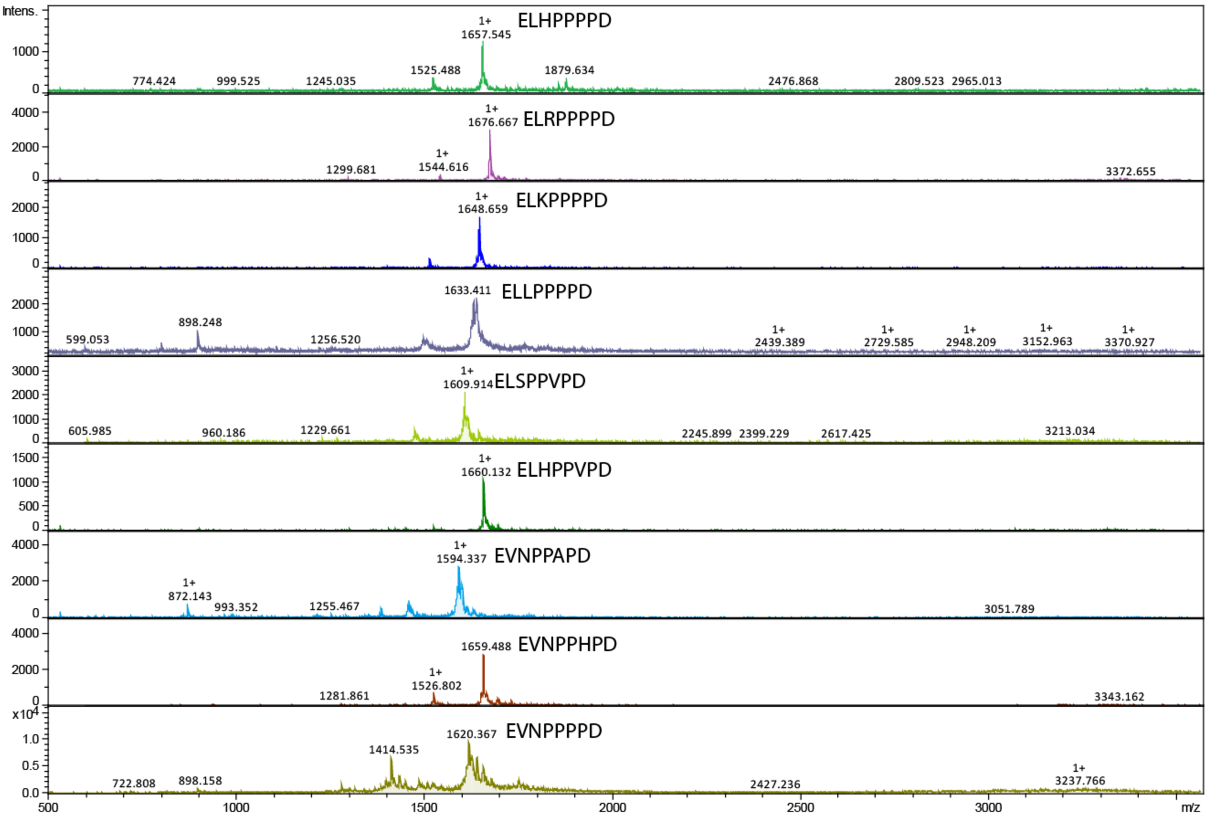

Supplementary Figure S8. Analysis of FRET-quenched peptides following synthesis by MALDI-ToF MS. MALDI-ToF MS was performed to assess the purity of the FRET-quenched peptides. The observed signals of the peptide mass minus a mass of ~132 Da are likely an artifact of the MALDI-ToF MS on peptides containing Lys(DABCYL). This mass loss is also observed in Supplementary Figure S9.

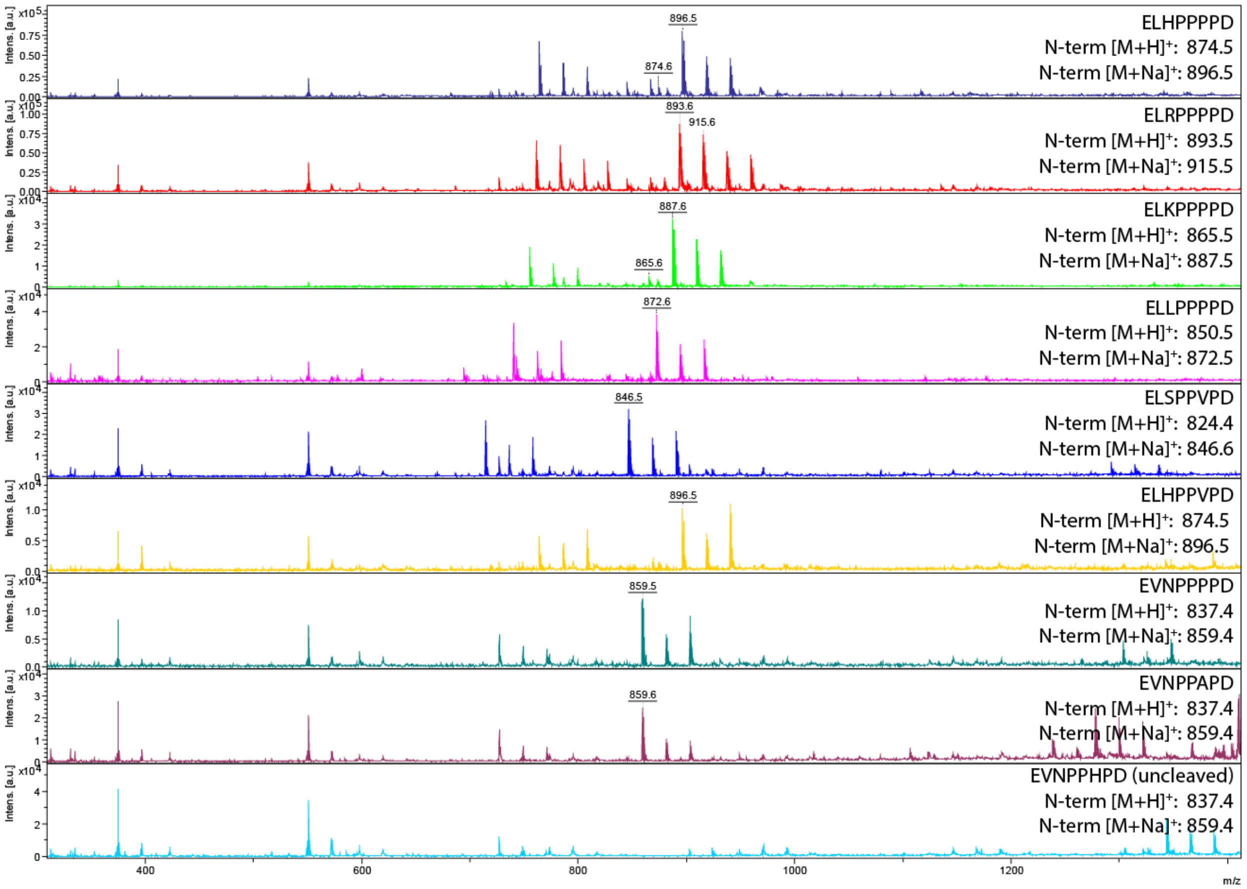

Supplementary Figure S9. Conformation of the Pro-Pro (P1-P1') cleavage site by MALDI-ToF MS. The masses of the expected N-terminal $[M+H]^+$ fragments and their sodiated counterparts ($[M+Na]^+$) following Pro-Pro cleavage are shown on the right. Due to the nature of the buffer, we mostly identified sodiated species. The peptide EVNPPHPD represents a very poor substrate and was therefore not cleaved by PPEP-3. Peaks with a mass loss of ~132 Da were observed, which are likely an artifact of the MALDI-ToF MS of peptides containing Lys(Dabcyl).

A

B

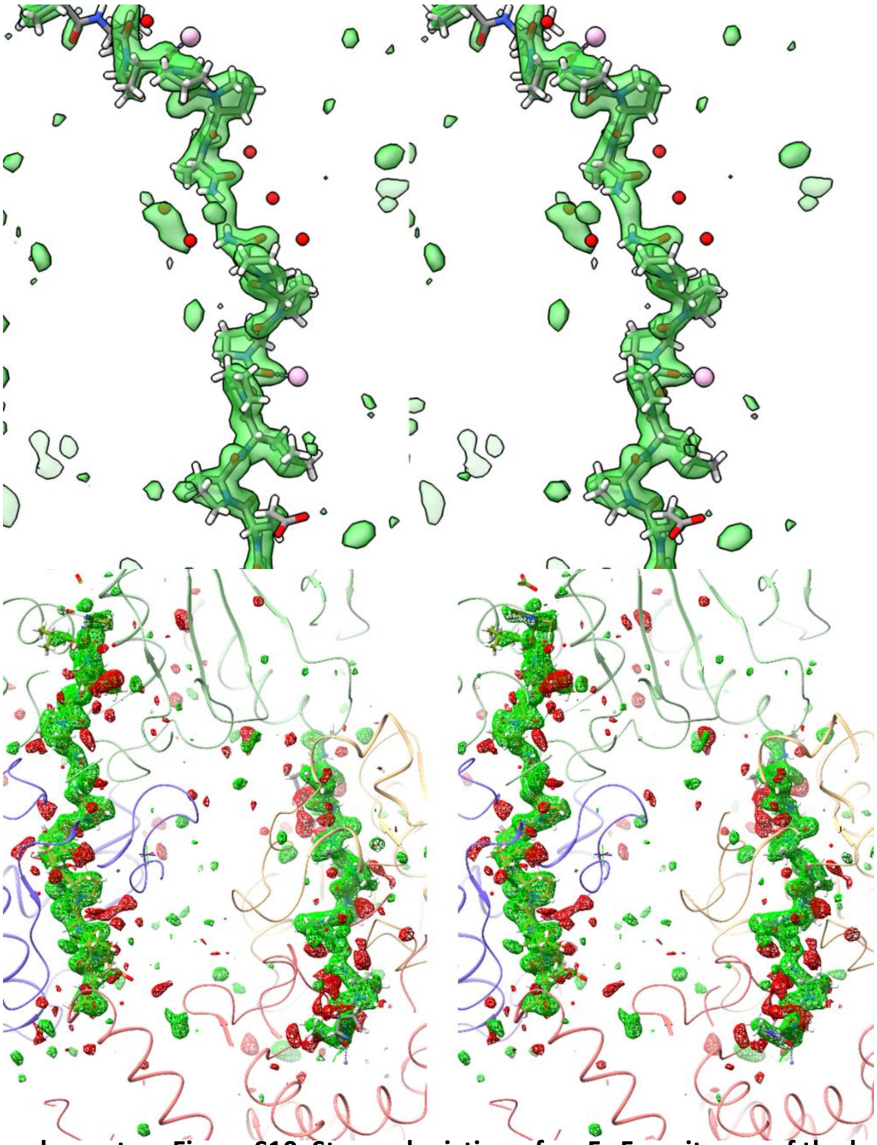

Supplementary Figure S10. Stereo depiction of an F_o-F_c omit map of the bound substrate peptide. (**A**) The difference map is shown as semitransparent surface at a contour level of +3.3 sigma in wall-eyed stereo representation. It depicts two substrate peptides adjacent in the asymmetric unit, oriented head-to-head, which are bound to two different PPEP-3 molecules in the asymmetric unit. Zinc ions (not omitted from map calculation) are shown as pink spheres. The map was created by deleting all substrate peptides from the refined coordinates, randomizing positional coordinates (RMS 0.3 Å) and randomizing B-factors followed by refinement with phenix.refine. Red spheres depict water molecules that were not omitted. (**B**) A similar difference map created in the same way as in **A**, but this time also omitting the zinc ions and showing a wider view of the asymmetric unit including positive (green) and negative (red) contour levels at +/- 3 sigma. The PPEP-3 backbone is shown in cartoon representation.

Supplementary Table S1: Data collection and refinement statistics

	wildtype*	E154A,Y190F*	Ac-EPLPPPP-CONH2 complex
PDB ID	9G0J	9G3T	9G5J
Wavelength (Å)	0.9677	0.8731	0.9677
Resolution range (Å)	44.64 - 1.55 (1.57 - 1.55)	62.81 - 1.598 (1.62 - 1.60)	47.71 - 2.00 (2.04 - 2.00)
Space group	P 41 21 2	P 41 21 2	P 21 21 21
Unit cell (Å, deg)	126.247 126.247 64.043 90 90 90	125.622 125.622 63.483 90 90 90	73.43 95.41 126.81 90 90 90
Total reflections	2,055,783 (61,588)	1,720,326 (34,341)	298,269 (16,447)
Unique reflections	143783 (4794)	128297 (4140)	113452 (6079)
Multiplicity	14.3 (12.8)	13.4 (8.3)	2.6 (2.7)
Completeness (%)	99.9 (98.2)	99.8 (96.2)	98.97 (99.47)
Mean I/sigma(I)	8.5 (0.72)	11.0 (1.19)	4.9 (0.86)
Wilson B-factor (Å2)	18.47	21.04	26.40
R-merge	0.2312(4.04)	0.135 (1.78)	0.165 (1.17)
R-meas	0.2404 (4.209)	0.1404 (1.896)	0.2041 (1.444)
R-pim	0.063 (1.16)	0.038 (0.65)	0.118 (0.83)
CC1/2	0.998 (0.102)	0.998 (0.242)	0.986 (0.232)
CC*	0.999 (0.431)	1 (0.624)	0.997 (0.614)
Reflections used in refinement	75414 (2477)	67412 (2139)	60271 (3182)
Reflections used for R-free	2619 (86)	2340 (79)	2602 (140)
R-work	0.174 (0.357)	0.158 (0.314)	0.191 (0.292)
R-free	0.199 (0.369)	0.180 (0.302)	0.232 (0.306)
Number of non-hydrogen atoms	3731	3768	7485
macromolecules	3440	3456	6983
ligands	49	58	33
solvent	242	254	469
Protein residues	421	421	865
RMS(bonds)	0.007	0.006	0.002
RMS(angles)	0.86	0.88	0.62
Ramachandran favored (%)	98.80	99.04	98.34
Ramachandran allowed (%)	0.96	0.72	1.66
Ramachandran outliers (%)	0.24	0.24	0.00
Rotamer outliers (%)	0.00	0.00	0.14
Clashscore	1.46	1.15	2.10
Average B-factor (Å2)	24.48	28.19	32.50
macromolecules	23.81	27.14	32.17
ligands	33.57	46.84	43.13
solvent	32.21	38.21	36.73

Values in parentheses refer to the outer resolution shell. Data collection statistics parameters like R-merge, R-meas or CC1/2 are defined in Diederichs *et al.*[4]. All reported CC1/2 values have been evaluated as statistically significant by the XDS package[5].

References

1. Claushuis, B., Cordfunke, R.A., De Ru, A.H., Van Angeren, J., Baumann, U., Van Veelen, P.A., Wuhrer, M., Corver, J., Drijfhout, J.W., Hensbergen, P.J., and Hensbergen, P.J. (2024). Non-prime- and Prime-side Profiling of Pro-Pro Endopeptidase Specificity Using Synthetic Combinatorial Peptide Libraries and Mass Spectrometry. *FEBS J.*, 2024.2003.2015.585006. 10.1101/2024.03.15.585006.
2. Gouet, P., Robert, X., and Courcelle, E. (2003). ESPript/ENDscript: Extracting and rendering sequence and 3D information from atomic structures of proteins. *Nucleic Acids Res. 31*, 3320–3323. 10.1093/nar/gkg556.
3. Laskowski, R.A., , Swindells, M.B., and (2011). LigPlot+: Multiple ligand-protein interaction diagrams for drug discovery. *J. Chem. Inf. Model. 51*, 2778–2786. 10.1021/CI200227U.
4. Diederichs, K., and (2016). Crystallographic Data and Model Quality. *Nucleic Acid Crystallography 1320*, 147–173. 10.1007/978-1-4939-2763-0_10.
5. Kabsch, W. (2010). XDS. *Acta Crystallogr D Biol Crystallogr. 66*, 125–132. 10.1107/S0907444909047337.
